# Mapping intellectual structures and research hotspots in the application of artificial intelligence in cancer: A bibliometric analysis

**DOI:** 10.3389/fonc.2022.955668

**Published:** 2022-09-22

**Authors:** Peng-fei Lyu, Yu Wang, Qing-Xiang Meng, Ping-ming Fan, Ke Ma, Sha Xiao, Xun-chen Cao, Guang-Xun Lin, Si-yuan Dong

**Affiliations:** ^1^ Department of Breast Surgery, The First Affiliated Hospital of Hainan Medical University, Haikou, China; ^2^ The First Department of Breast Cancer, Tianjin Medical University Cancer Institute and Hospital, National Clinical Research Center for Cancer, Key Laboratory of Cancer Prevention and Therapy, Key Laboratory of Breast Cancer Prevention and Therapy, Tianjin Medical University, Ministry of Education, Tianjin’s Clinical Research Center for Cancer, Tianjin, China; ^3^ International School of Public Health and One Health, Heinz Mehlhorn Academician Workstation, Hainan Medical University, Haikou, China; ^4^ Department of Orthopedics, The First Affiliated Hospital of Xiamen University, School of Medicine, Xiamen University, Xiamen, China; ^5^ Thoracic Department, The First Hospital of China Medical University, Shenyang, China

**Keywords:** cancer, application, research hotspots, bibliometric analysis, ai

## Abstract

**Background:**

Artificial intelligence (AI) is more and more widely used in cancer, which is of great help to doctors in diagnosis and treatment. This study aims to summarize the current research hotspots in the Application of Artificial Intelligence in Cancer (AAIC) and to assess the research trends in AAIC.

**Methods:**

Scientific publications for AAIC-related research from 1 January 1998 to 1 July 2022 were obtained from the Web of Science database. The metrics analyses using bibliometrics software included publication, keyword, author, journal, institution, and country. In addition, the blustering analysis on the binary matrix was performed on hot keywords.

**Results:**

The total number of papers in this study is 1592. The last decade of AAIC research has been divided into a slow development phase (2013-2018) and a rapid development phase (2019-2022). An international collaboration centered in the USA is dedicated to the development and application of AAIC. Li J is the most prolific writer in AAIC. Through clustering analysis and high-frequency keyword research, it has been shown that AI plays a significantly important role in the prediction, diagnosis, treatment and prognosis of cancer. Classification, diagnosis, carcinogenesis, risk, and validation are developing topics. Eight hotspot fields of AAIC were also identified.

**Conclusion:**

AAIC can benefit cancer patients in diagnosing cancer, assessing the effectiveness of treatment, making a decision, predicting prognosis and saving costs. Future AAIC research may be dedicated to optimizing AI calculation tools, improving accuracy, and promoting AI.

## Introduction

Artificial intelligence (AI) uses science and technology to simulate human intellectual skills and solve medical problems involving complex genetic defects such as cancer (1). Artificial intelligence is the ability to use computers to perform tasks, which are usually associated with humans (2). The primary health problem studied in AI research is cancer (3). The rapid development of artificial intelligence in the past has contributed much to basic research, diagnosis and treatment of cancer, compensating for the fact that the human brain can only manage a large amount of information in a limited amount of time. Certain cancers have a relatively poor prognosis, detected late and the effectiveness of treatment is often greatly compromised. AI with deep learning has improved the diagnostic ability to detect esophageal cancers, including squamous cell carcinoma and adenocarcinoma, leading to a better prognosis in the future (4). In recent years, deep learning (DL) has received much attention because it uses multi-level abstraction learning methods to process input data and detect complex structures automatically (5). AI support provides radiologists with interactive decision support, computerized detection of abnormal conventional lesion markers, and an examination-based cancer likelihood score (6). As a result, cancer detection rates for doctors on mammograms have improved without additional work time.

Convolutional neural networks can be effectively used for speech recognition, image processing, and other tasks in which artificial neurons respond to some of the surrounding units, such as feed-forward neural networks. The convolutional neural networks (CNN) for detecting gastric cancer with AI can process a large number of stored endoscopic images in a concise time and has clinically relevant diagnostic capabilities (7). It assists in routine clinical practice, greatly improving the efficiency of the endoscopist and enhancing the accuracy of the report (8). For example, the Gastrointestinal Artificial Intelligence Diagnostic System has high diagnostic accuracy in detecting cancers of the upper gastrointestinal tract. Its sensitivity is similar to that of an expert endoscopist and superior to a non-expert endoscopist. In the meantime, artificial intelligence has been used to assess survival and prognosis outcomes for patients with ovarian and cervical cancer (9, 10). The tumor microenvironment (TME) plays an important role in tumorigenesis and progression. Novel techniques in artificial intelligence (AI) can help determine areas of therapeutic need, enhance clinical trial interpretation, identify novel targets, and generate accurate predictions that are impossible with traditional statistical techniques (11).AI is used to assess TME, prognosis and the benefit of adjuvant chemotherapy in patients after radical gastric cancer surgery and is a valuable addition to the current TNM staging system (12).

In bibliometrics, current research and development in a field can be analyzed quantitatively and qualitatively. This tool provides practitioners, students, researchers, and hobbyists with an objective assessment of a specific field’s contribution and an understanding of current and future trends in research in the field by visually analyzing the countries, authors, institutions, journals, citations, and topics related to the field and extracting the corresponding conclusions. The latest bibliometric analysis of cancer and AI, however, lacks a comprehensive overview. Therefore, a review and summary of articles in this area would help readers get a better understanding of AAIC developments over the past decade and what to expect in the future.

## Materials and methods

### Data sources and search strategies

**Figure 1:**
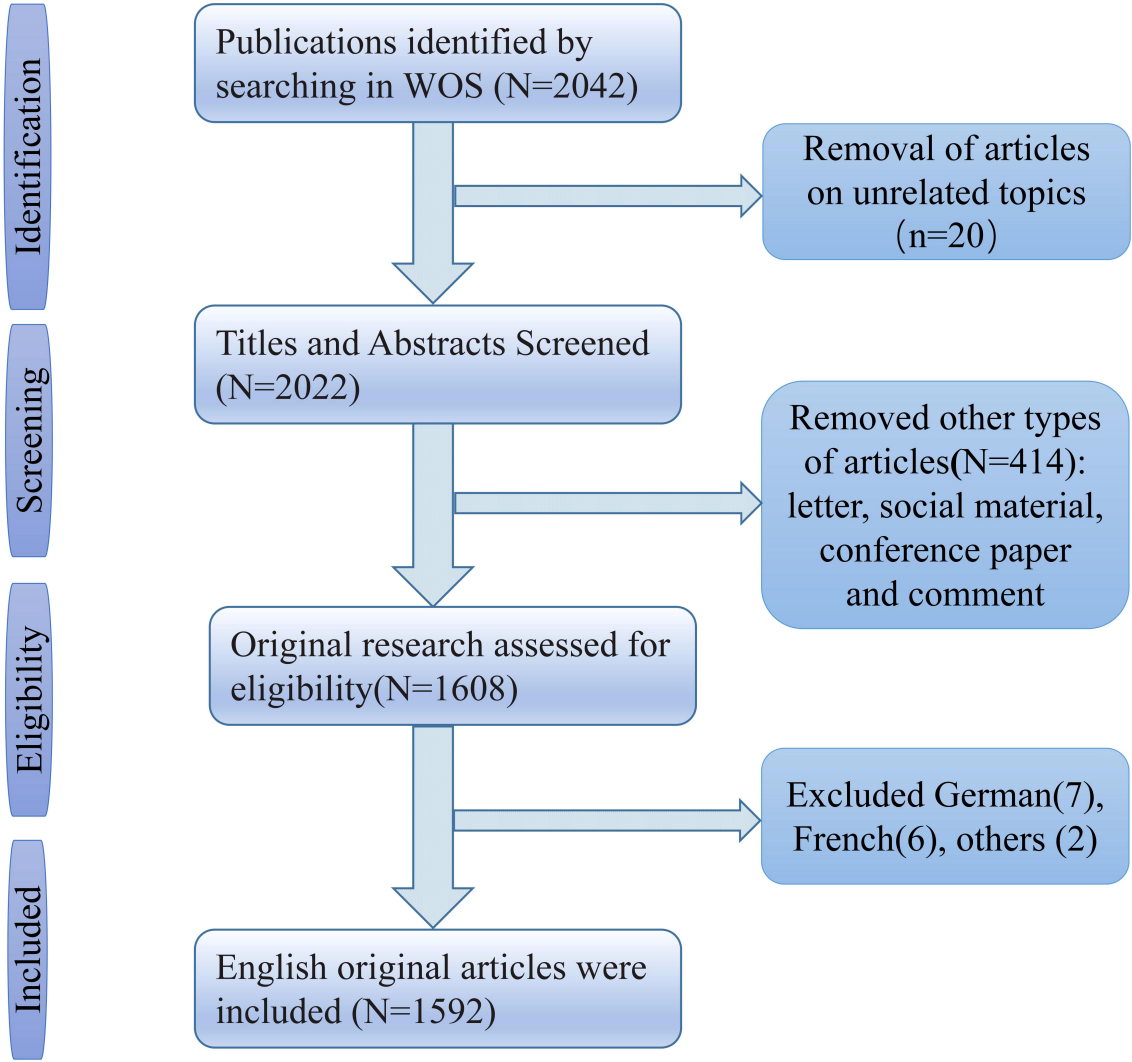
Flow diagram of data extraction of AAIC.

We used the Web of Science database for our search, often used for bibliometric analyses because of its strict assessment of publications and high-quality literature. All data were retrieved and exported from 1 January 1998 to 1 July 2022. The search strategy used the following keywords: (“artificial intelligence*” or “Computational Intelligence*” or “Machine Intelligence*” or “Deep learning” or “Convolutional Neural Network*” or “DL” or “CNN”) AND (“cancer*” or “carcinoma*” or “neoplasm*”). Publications authored in English and original research articles fulfilled the inclusion requirements. The following articles are excluded: letters, social material, conference papers, and reviews. Two independent researchers extracted relevant data from screened articles, including titles, keywords, authors, institutions, journals, countries and regions, and total citations. The flow chart is listed in **Figure 1**.

### Bibliometric analysis

We utilized CiteSpace, R language, and VOSviewer to create data tables and structured knowledge graphs for visualization research linked to the publications, countries, h-index (a valid and reliable indicator for academic assessment) (13), institutions, journals, authors, citations, co-occurrence status, hotspots, and keywords. CiteSpace is based primarily on co-citation analysis and pathfinder network scaling to analyze the literature on a particular subject so that users can identify the critical developments and knowledge turning points in the history of the discipline (14–16). VOSviewer is a tool for creating maps based on network, bibliographic, or text data (17–20). R-based Biblioshiny app provides a web interface for bibliometrics (https://bibliometrix.org/) (21–24). The frequency with which a tag or circle appears increases its size. The label and circle for an item become more prominent. The proximity of two items in the visual knowledge graph indicates the frequency with which they occur jointly, shown by the strength of the linkages between the nodes (circles).

Using the software “gCLUTO” version 1.0 (http://glaros.dtc.umn.edu/gkhome/cluto/gcluto/download), a binary matrix with important standard terms as rows and source articles as columns was created in BICOMB for further dichotomous clustering (25–28). Parameters for dichotomous clustering in gCLUTO were adjusted to match those recommended for dichotomous cluster analysis. Clustering was performed using repeated dichotomies, and cosines were used to measure similarity (29). Results from a dichotomization of the publicly accessible main keyword-source article matrix were shown using ideal mountain visualization and matrix visualization. Semantic connections between hotspot words and the content of sample articles in each cluster were used to generate the AAIC research hotspots’ core framework, which was then examined.

## Results

### Primary information about the collection

The time span is from 1998 until 2022. In our investigation, the total number of documents reached 1592. From 1998 through 2018, there was a modest yearly rise, with 41 papers published in 2018. Since then, there has been a significant growth. In 2021, the number of articles published surpassed the previous year’s total of 583. Since 2019, the number of articles produced within a year has increased dramatically.

**Figure 2:**
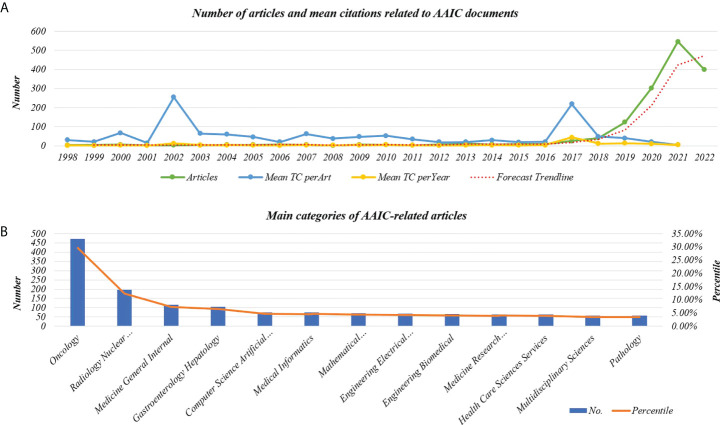
Line graph of information about AAIC documentation. **(A)** The number of articles and mean citations related to AAIC. **(B)** Main categories of AAIC-related articles. TC, Total citations.

The average total citation (TC) per article is 15.98 and the average total citations (TC) per year is 4.13. The number of articles and mean citations related to AAIC are shown in **Figure 2A**. There are 10130 authors involved in the field. The average number of authors per document is 6.36, and the keyword plus is 2877. The author’s keyword is 3371. The categories of documents linked to AAIC are shown in **Figure 2B**. The main categories were Oncology (29.585%), Radiology Nuclear Medicine Medical Imaging (12.312%), Medicine General Internal (7.224%), Gastroenterology Hepatology (6.533%), and Computer Science Artificial Intelligence (4.711%).

### Distribution and cooperation characteristics of countries

**Figure 3:**
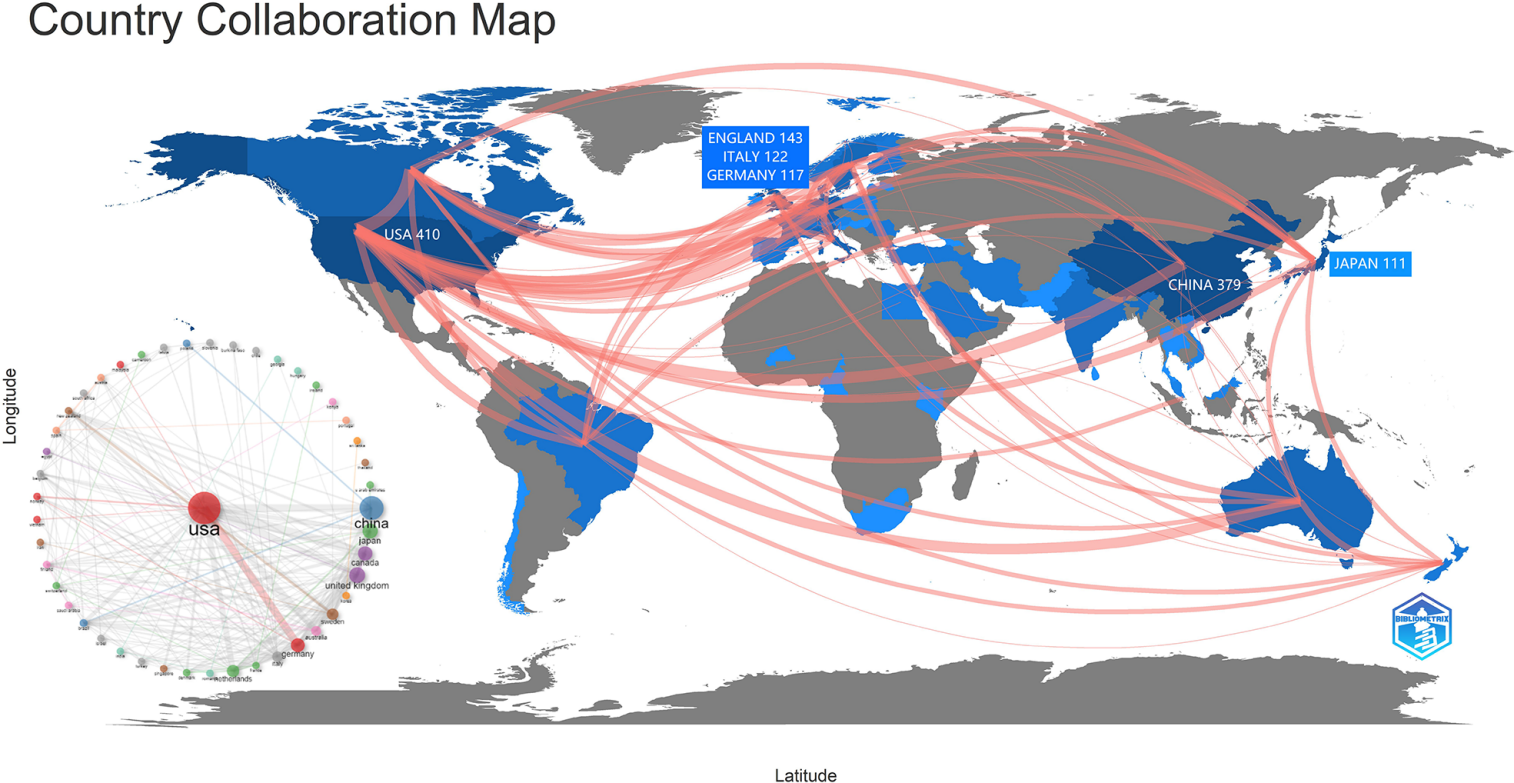
The distribution and cooperation characteristics of countries.

**Figure 3** depicts a map showing the distribution and cooperation of nations. The amount of red lines corresponds to the level of regional collaboration. The intensity of blue indicates the quantity of papers issued by the government. The United States had the greatest number of publications (n= 410), followed by China (n= 379), England (n= 143), Italy (n= 122) and Germany (n= 122). (117). Many connections exist between nations. AAIC is a global hotspot of concern. **Figure 3** reveals that the United States has the most links with other nations, suggesting that the AAIC based on the United States is gaining prominence, followed by China.

### Author, journal, and institution analysis

For the development of AI, 2850 institutions from around the world participated. Of the 1952 publications, 196 were produced by five institutions: HARVARD UNIVERSITY published 43 (2.701%); UNIVERSITY OF TEXAS SYSTEM (America) published 38 (2.387%); UNIVERSITY OF LONDON, published 37 (2.324%); and RUPRECHT KARLS UNIVERSITY HEIDELBERG, published 36 (2.261%). The top three of them are from the United States.

During the study period, 594 scientific magazines published articles about AAIC. The five journals that published the most articles about AAIC were CANCERS, with 89 articles (5.590%); FRONTIERS IN ONCOLOGY, with 63 (3.957%); the DIAGNOSTICS, with 31 (1.947%); EUROPEAN RADIOLOGY with 25 (1.570%).

Analyzing the distribution of core authors allows for a more comprehensive assessment of academic exchanges, cooperation and research development. In **Table 1**, 5 authors who published more than 14 papers are listed. Brinker, TJ was the most prolific author in AAIC, having published eighteen articles (1.131%); Tada T, published eighteen articles (1.131%); Li J, published fifteen articles (0.942%); Kather JN and Wang J published fourteen articles (0.879%); Wang J, and Tada T published every five articles (1.96%).

**Table 1:** Top 5 authors based on the number of documents related to AAIC.

Author	Number of Documents	Total Citation Frequency	Average Citation Frequency per Year	Average Citation Frequency per Paper	h-index	Time	Country
Brinker, TJ	18	312	78	17.33	9	2019-2022	Germany
Mann, R.M	18	789	159.6	44.33	12	2018-2022	Japan
Li, J	15	125	31.25	8.33	4	2019-2022	China
Tada T	14	156	52	11.14	6	2020-2022	Germany
Wang J	14	88	29.33	6.29	5	2019-2022	China

Notably, two of the top five academics are Chinese and two are German. The publishing period is focused between 2019 and 2021, which coincides with the AAIC’s publication spike, showing that they are the primary writers throughout the era of expansion and intensification. In addition, based on the average number of citations per paper, Brinker, TJ, Mann, R.M., and Tada T are more often mentioned than the other four writers, indicating that their publications are of more importance and are more well known. They have not yet developed a core group of authors.

**Figure 4:**
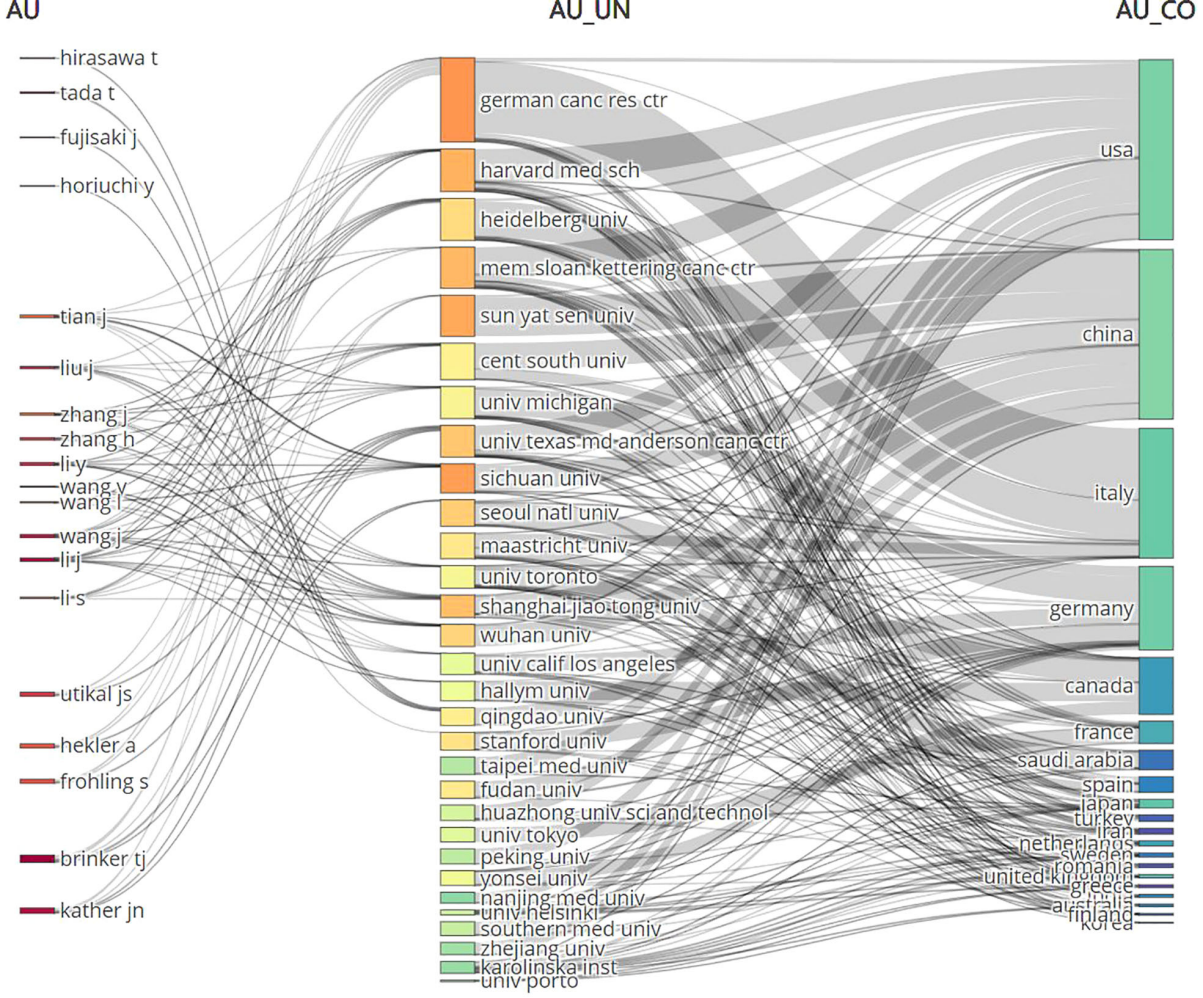
The three-fields plot of authors, journals, and countries. AU, Author; UN, Unit; CO, Country.

These three components, which consist of authors, journals, and nations, are represented by a “three-field plot.”(**Figure 4**). The three elements are linked in grey, with the author’s name first, followed by the institution, and then each author’s link to the country they published their paper. The size of each rectangle in each list represents the number of papers associated with that element.

### Analysis of references and citations

**Figure 5:**
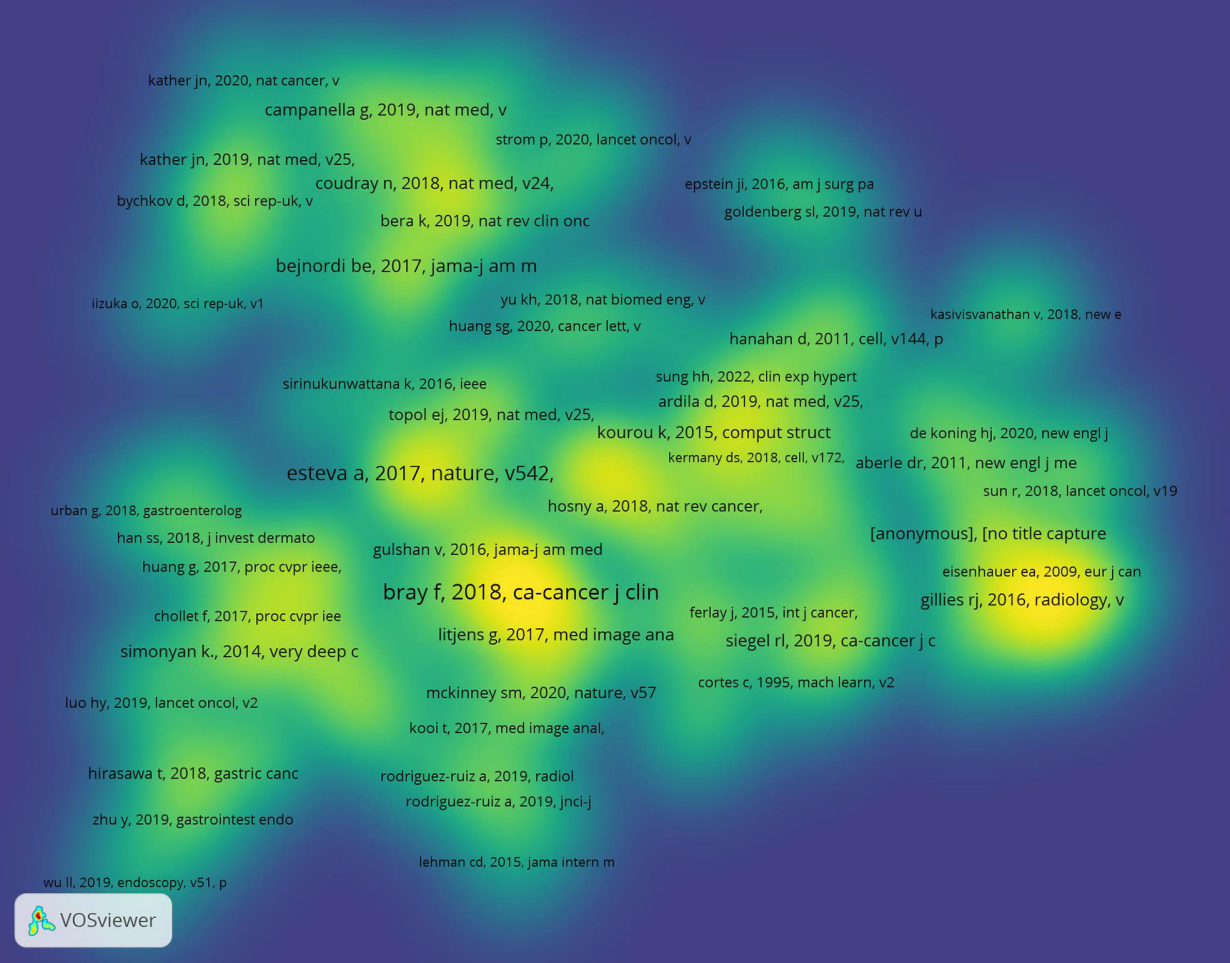
The density map of citation visualization.

**Table 2** is a listing of the top 10 most-cited articles. The highest- and lowest-cited articles have a total of 4533 and 177 citations, respectively. The vast majority of these 10 pieces came from the United States and were published between 2017 and 2020. **Figure 5** displays a depiction of citation density. According to the literature’s strength of application, the closer the hue is to yellow, the greater the density.

**Table 2:** The top 10 most cited articles.

Rank	Title	Author	Journal	Year	Citation	IF(2021)	Country
1	Dermatologist-level classification of skin cancer with deep neural networks	Esteva, A	NATURE	2017	4,533	69.504	USA
2	Serum protein fingerprinting coupled with a pattern-matching algorithm distinguishes prostate cancer from benign prostate hyperplasia and healthy men	Adam, BL	CANCER RESEARCH	2002	745	13.312	USA
3	International evaluation of an AI system for breast cancer screening	McKinney, SM	NATURE	2020	586	69.504	USA
4	Artificial intelligence in cancer imaging: Clinical challenges and applications	Bi, WL	CA-A CANCER JOURNAL FOR CLINICIANS	2019	417	286.13	USA
5	Predicting cancer outcomes from histology and genomics using convolutional networks	Mobadersany, P	PROCEEDINGS OF THE NATIONAL ACADEMY OF SCIENCES OF THE UNITED STATES OF AMERIC	2018	309	12.779	USA
6	Application of artificial intelligence using a convolutional neural network for detecting gastric cancer in endoscopic images	Hirasawa, T	GASTRIC CANCER	2018	288	7.701	Japan
7	Diagnosis of breast cancer using elastic-scattering spectroscopy: preliminary clinical results	Bigio, IJ	JOURNAL OF BIOMEDICAL OPTICS	2000	241	3.758	USA
8	Genetics and biology of prostate cancer	Wang, GC	GENES & DEVELOPMENT	2018	205	12.89	USA
9	Somatic Mutations Drive Distinct Imaging Phenotypes in Lung Cancer	Velazquez, ER	CANCER RESEARCH	2017	192	13.312	USA
10	Predicting response to cancer immunotherapy using noninvasive radiomic biomarkers	Trebeschi, S	ANNALS OF ONCOLOGY	2019	177	51.769	Netherlands

### Keywords and thematic evolution

**Figure 6:**
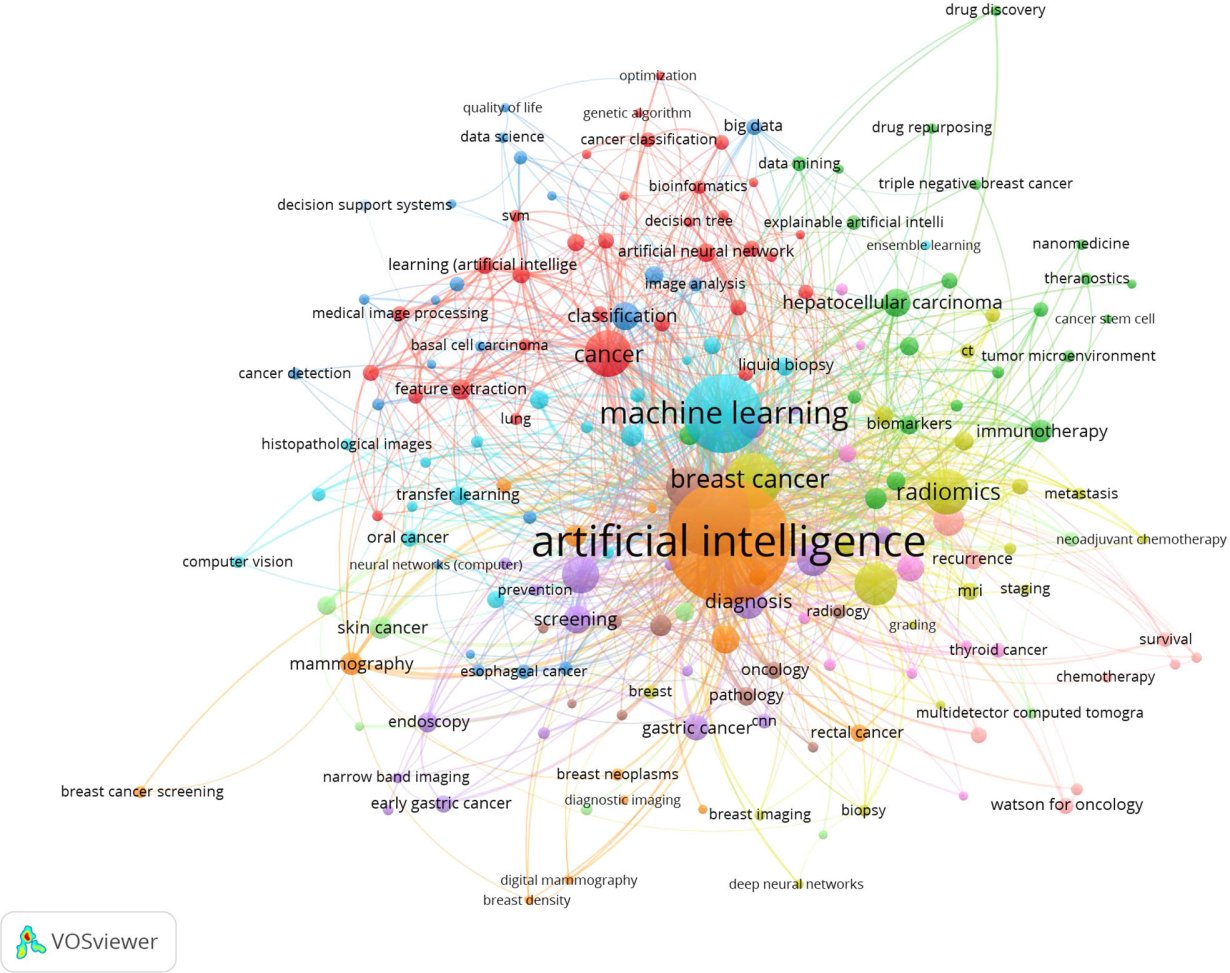
The co-occurrence map of keywords.

**Figure 7:**
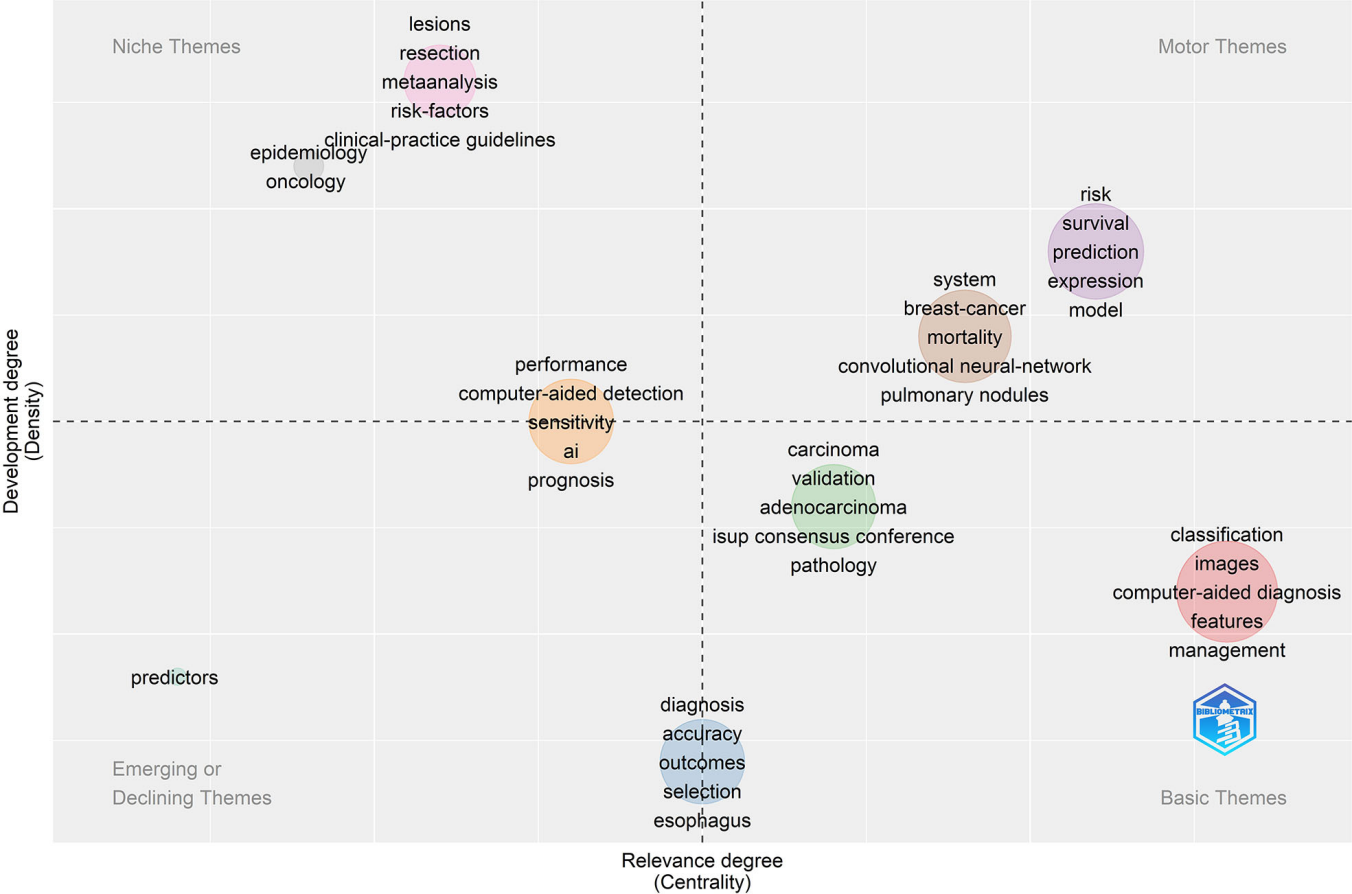
The thematic map of keywords.

Keywords frequently reflect an article’s core and the main content. The cluster map reflects research hotspots effectively. In the keywords co-occurrence map (**Figure 6**), larger nodes exhibited larger keyword weights, signifying the frequency of occurrence of the keyword. The concatenation of nodes represents the co-occurrence of keywords. Here, the top significant keywords were AI, classification, diagnosis, carcinoma, risk, survival, performance, system, images, prediction, validation, computer-aided detection, breast cancer, expression, mortality, lesions, computer-aided diagnosis, convolutional neural network, features, management, and neural-networks. We evaluated the evolution of AAIC trends utilizing thematic map (**Figure 7**). The abscissa represents the centrality correlation coefficient, while the ordinate represents the growth of buzzwords. The motor themes, which are central and well-developed, are in the upper right quadrant, while the isolated themes, the peripheral themes, and the fundamental themes are all located in the bottom right. The root issues are with diagnosis, prognostic staging, and imaging. Cancer risk assessment and prognostic markers are essential topics since they indicate the current limits of knowledge in the AAIC area. Due to their potential future success as primary driving themes, these technologies have a lot of potential.

**Figure 8:**
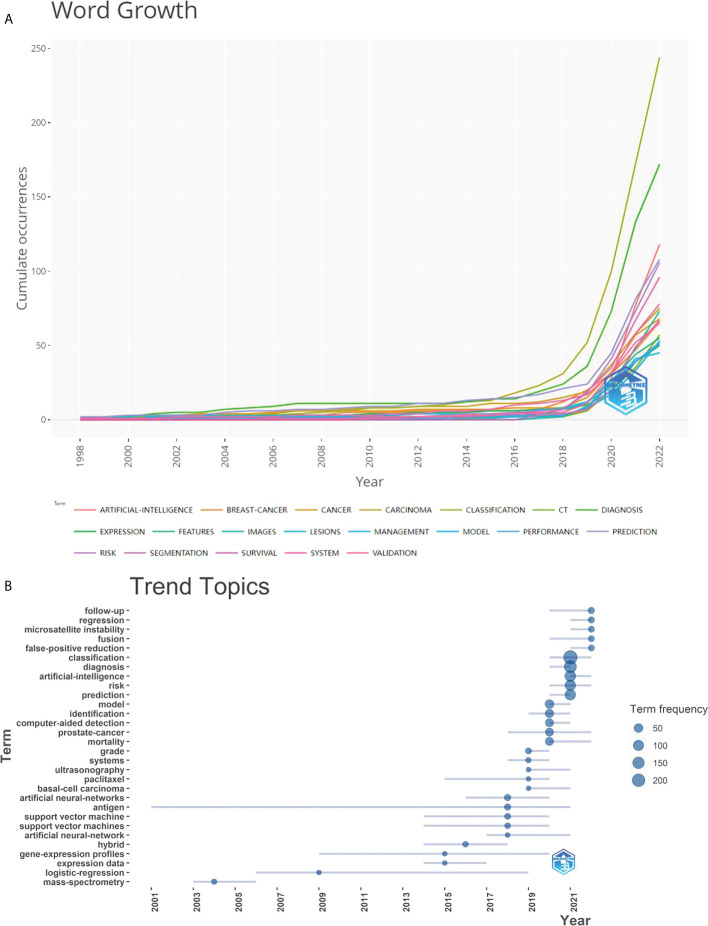
Graphs of keyword growth and subject line trends. **(A)** The map of cumulative growth of core keywords over time; **(B)** The Graph of trend changes in subject terms over time.

**Figure 8** displayed a graph indicating the development of keywords and the tendencies in subject lines. From the total growth map of keywords (**Figure 8A**) and the terms trend chart (**Figure 8B**), we can discern that classification, diagnosis, carcinogenesis, risk, and validation are emerging themes aggressively, particularly in recent years.

### Hotspot cluster analysis

**Figure 9:**
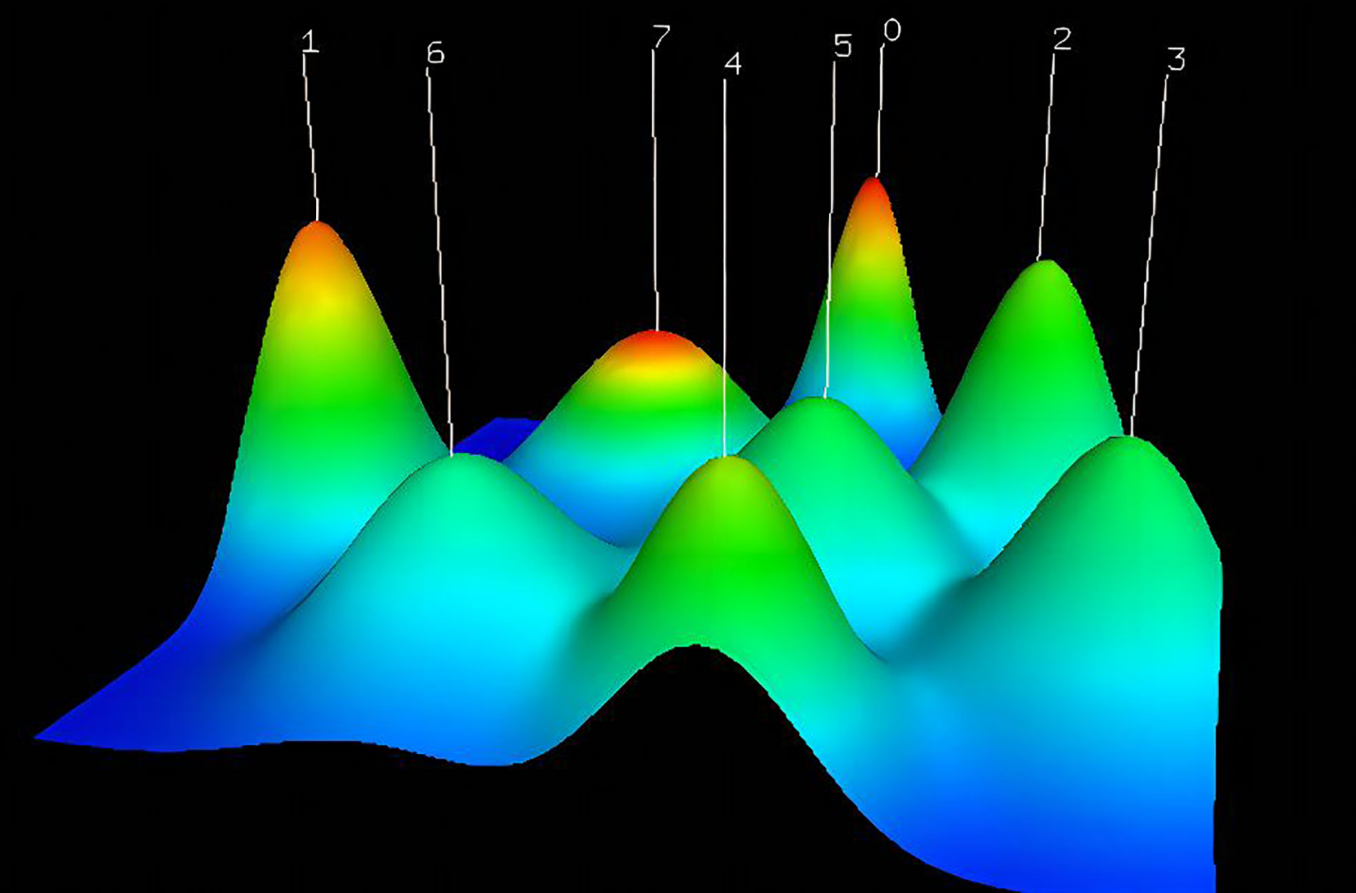
The visualized mountain map of the keywords: Cluster 0: AI for diagnosis of gastric cancer and assessing tumor microenvironment; Cluster 1: AI for skin cancer diagnosis; Cluster 2: AI for assessing cancer prognosis; Cluster 3: AI models for assessing treatment response; Cluster 4: AI in lung cancer; Cluster 5:AI for Early Detection of Cancer; Cluster 6: AI for cancer prediction; Cluster 7: AI for breast cancer and cost analysis.

This paper was made more visually appealing and analytically focused by avoiding unnecessary keywords, standardizing capitalization, combining synonyms, and selecting keywords that appeared at least three times for matrix extraction and cluster analysis. Numbers are highlighted to indicate their corresponding clusters on the graphical mountain range (**Figure 9**). Its volume is proportional to the number of clustered keywords, and its height is proportional to the similarity within classes.

This mountain map comprises five colors: red, yellow, green, light blue, and dark blue, which reflect the rising standard deviation of intraclass similarity. A red peak indicates little intraclass variation. In contrast, if the color of the peak is blue, intraclass variance is substantial. Using the close distance between the two peaks, the similarity between the two clusters was determined. The various clustering approaches produced eight peaks that were somewhat independent and widely dispersed, suggesting successful clustering.

**Figure 10:**
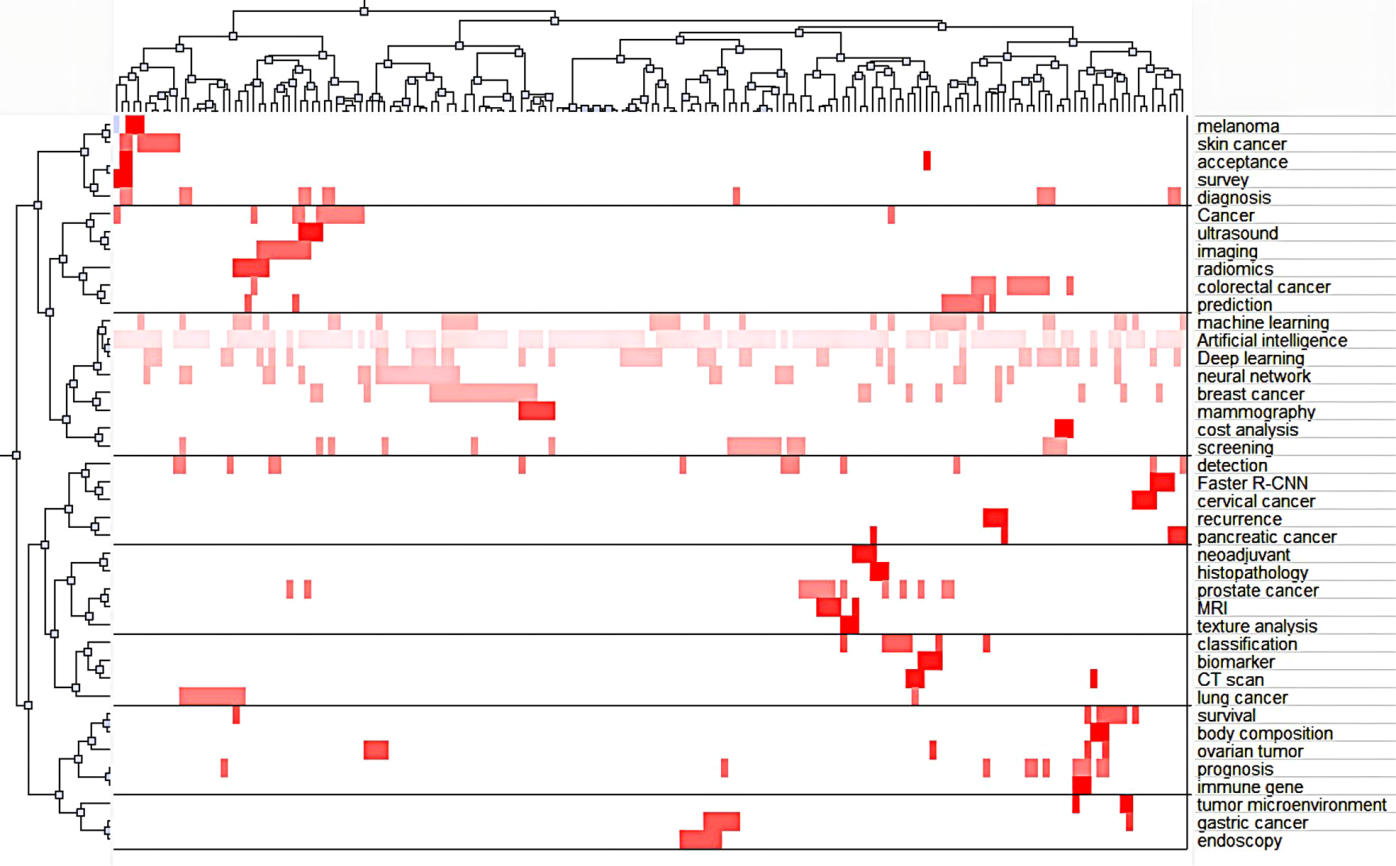
The visualized heat map linked to data matrix of keywords.

**Figure 10** is a heat map visualization, where columns indicate high-frequency terms, rows represent literary works, and the red hues reflect the matrix of values in the original data based on color depth. The graph’s white regions represent values very close to zero. Greater prominence in the red indicates more often occurring terms in the value clustering tree. Using a series of dichotomous cluster analyses to establish the relationship between articles and buzzwords, we were able to identify eight active lines of inquiry within the AAIC domain:

Cluster 0: AI for diagnosis of gastric cancer and assessing tumor microenvironment
Cluster 1: AI for skin cancer diagnosis
Cluster 2: AI for assessing cancer prognosis
Cluster 3: AI models for assessing treatment response
Cluster 4: AI in lung cancer
Cluster 5:AI for early detection of cancer
Cluster 6: AI for cancer prediction
Cluster 7: AI for breast cancer and cost analysis

## Discussion

In recent years, there has been substantial research interest in the expanding application of artificial intelligence (AI) in health and medicine (30). The number of publications has climbed dramatically over the last three years, notably in 2021, which surpassed the total of the preceding eight years combined. During the first two quarters of 2022, 388 publications were produced. By constructing a forecast curve, we estimate that 480 articles will be published in 2022 (**Figure 2A**). This increase may be due to the fast development of the application of AI in the field of cancer, and scholars pay more attention to AAIC. Therefore, AAIC research has been divided into the slow development stage (1998-2017) and the rapid development stage (2018-2022). Through keyword research, we observed that AAIC throughout the slow growth phase were focused on diagnostics and imaging. AAIC has been progressively utilized to predict survival, as well as to aid in diagnosis, surgery, cancer classification, validation, and early diagnosis during the last three years. The applications are becoming more prevalent. In the future, researchers may explore at forecasting survival, predicting disease risk, and utilizing AI to identify lung cancer nodules. Although AI has gradually changed the traditional treatment, it still needs time and many challenges to mature (31).

By analyzing the number of papers, authors and institutions, it is found that the AAIC centered on the United States is becoming more and more prominent. In terms of citation, the most referenced papers were discovered to emerge frequently from 2017 to 2020, primarily from the United States. Once again, it shows that the United States has made great contributions to AAIC.

Furthermore, we discover that academics from different countries often interact with one another, and that the worldwide collaboration led by the United States is dedicated to the advancement and practical implementation of AAIC. A great number of AAIC-related organizations and journals originate in China, England, and other nations, demonstrating their significant contributions. This may be attributed to the strong scientific and technical foundations as well as the thriving economy in these nations. **Table 3** highlights the 10 leading funding agencies in terms of the quantity of papers received. The United States government was the most supportive. AAIC’s progress has been inconsistent because to differences in economic development. Many low-income nations have failed to engage extensively in AI technologies in the healthcare industry due to insufficient healthcare resources (3, 32). As a consequence of excellent policy and budgetary support, developed countries create around 85% of research output (33, 34). However, due of their large population, emerging nations have a bright future in terms of AI (35).

**Table 3:** Top 12 Funding agencies based on the number of documents.

Funding Agencies	Record Count	% Of 252	Country
National Natural Science Foundation of China Nsf	147	9.234%	China
United States Department of Health Human Services	128	8.040%	USA
National Institutes of Health Nih Usa	127	7.977%	USA
Nih National Cancer Institute nci	74	4.648%	USA
European Commission	55	3.455%	France
Uk Research Innovation Ukri	26	1.633%	England
Ministry Of Education Culture Sports Science and Technology Japan Mext	22	1.382%	Japan
Japan Society for The Promotion of Science	19	1.193%	Japan
National Key R D Program of China	18	1.131%	China
Medical Research Council Uk Mrc	17	1.068%	England

Although we analyzed the top five authors, magazines and institutions, their influence on AAIC is not so great because they have less articles. For example, Brinker, TJ from German Cancer Res Ctr of Germany was the most prolific author in AAIC, but his articles are not in the top ten cited articles. It demonstrates that influential authors, magazines, and institutions have yet to emerge.

Through cluster analysis and research of high-frequency keywords, AI plays a significant role in the prediction, diagnosis and treatment (36–38). Predicting cancer risk and prognosis may have great room for development in AAIC (39). The application of artificial intelligence in predicting the risk of liver metastasis of colorectal cancer has been realized (40). The AAIC has highlighted eight of the most active research fields.

### Cluster 0: endoscopic device systems and gastric cancer

Several medical sectors, like gastrointestinal endoscope, have adopted AI using deep learning technology to recognize and distinguish library pictures (41). The discipline of gastrointestinal endoscopy includes the endoscopic diagnosis and prognosis of numerous digestive illnesses utilizing image processing with the use of a variety of endoscopic equipment systems. The gastric cancer diagnosis algorithm based on artificial intelligence demonstrates a reasonably good diagnostic accuracy, distinguishing and diagnosing non-neoplastic lesions such as benign gastric ulcer and dysplasia (42). Following radical gastrectomy, patients can utilize AI to assess TME, prognosis, and the efficacy of adjuvant treatment (12). As such, it is an adjunct to conventional care for patients with gastric cancer.

### Cluster 1: AI for skin cancer diagnosis and survey

AI is currently applied in the evaluation of skin cancer. Dermatologists can construct these models for use in skin cancer screening and diagnosis with the use of available datasets, deep machine learning pictures, or photos of gross lesions accompanied by a skin cancer or melanoma diagnosis (43). Comparing the visual diagnostic abilities of artificial intelligence and three dermatologists for skin cancer lesions, it was observed that the artificial intelligence model could better differentiate melanoma from non-melanoma, proving the model’s efficacy in discriminating pictures (44). Although AAIC is emerging in the field of skin cancer, it faces two problems: data set bias and problems in the technical application; the accepted population in translation into clinical practice is generally under the age of 35 (45). So AAIC needs more interpretability and engagement.

### Cluster 2: AI for assessing cancer prognosis

Indirectly, AAIC can evaluate cancer prognosis and give a reference for therapeutic efficacy. Algorithms using multilayer perceptron artificial neural networks, radial basis functions, gene set enrichment analysis (GSEA), and conventional statistics accurately predicted MCL overall survival. They identified survival-predicting genes in a sizeable pan-cancer cohort (9). The data-driven relationship between immune cell composition in the tumor microenvironment (TME) and >=5-year survival in breast cancer patients was investigated using an interpretable artificial intelligence (XAI) model to accurately predict clinical outcomes, thereby designing innovative strategies to cure cancer (11).

### Cluster 3: AI models for assessing treatment response

AI has greatly aided us in determining the response of cancer therapy. AI-based imaging after adjuvant chemotherapy allows for more accurate treatment evaluation. AI models based on textural aspects of MR images of patients with locally advanced rectal cancer (LARC) may aid in identifying patients who will achieve complete pathological remission (PCR) after treatment and those who will not respond to therapy (NR) at an early stage (46). AI quantification of histopathological response to neoadjuvant therapy (NAT) can be used to guide adjuvant therapy and compare the efficacy of neoadjuvant regimens (47). A modified U-network with DenseNet161 encoder yields the most excellent average segmentation accuracy. As a result, AI-based evaluation of residual tumor burden is achievable (48).

### Cluster 4: AI in lung cancer

The application of artificial intelligence in lung cancer is mainly in screening and diagnosis (49). The primary goals of screening are to identify high-risk populations and automatically detect lung nodules. Imaging, pathology, and genetic diagnoses are all types of diagnoses. Currently, the obstacles to using AI in lung cancer primarily focus on the interpretability of AI models, privacy, and limited annotated datasets; breakthroughs in interpretable machine learning, transfer learning, and federated learning may overcome this dilemma.

Furthermore, the precision of diagnosis and screening need to be constantly enhanced. Deep learning and machine learning techniques, for example, may accomplish high-precision automated characterization and categorization of nodules and are predicted to become an advanced lung cancer screening approach in the future (50). There is still a need to create an effective validated model for the merging of artificial intelligence with radiography to assist clinicians in making treatment choices (51, 52).

### Cluster 5: AI for early detection of cancer

Artificial intelligence has great potential in the early detection of cancer. Utilizing an algorithm and establishing a model based on artificial intelligence, can improve the sensitivity of radiologists in breast cancer screening and detection (53, 54).

Gigabytes of sequencing data from liquid biopsies of several patients are searched using artificial intelligence to assess disease-related traits and diagnose cancer with high specificity (55). Prostate detection using AI is achieved by designing certain features of tissue. Histopathological pictures are used in radiology to classify tumors as benign or malignant by extracting relevant characteristics or information (56).

### Cluster 6: AI for cancer prediction

Individualized treatment decisions may benefit from the use of an AI survival prediction system. Synthesized intelligence worked on a novel anastomotic leakage prediction model for colorectal double stapler (DST) anastomosis (57). To anticipate recurrence, the automatic artificial intelligence program “prediction one” (Sony Network Communications Inc.) is employed, which enhances accuracy over the old approach (58). It is critical to detect and treat oral cancer as soon as possible to improve survival rates. Oral cancer risk prediction using an artificial neural network trained on individual-level data on risk factors, systemic medical problems, and clinic-pathological aspects (59).

Presently, the prediction models of tumor prognosis, therapeutic toxicity, and pathological results have been established (60). However, exploratory studies are promising, and validation studies that demonstrate consistency, reproducibility and prognostic impact are still needed.

### Cluster 7: AI for breast cancer and cost analysis

Breast cancer is a severe threat to women’s health. Early detection of breast cancer can significantly improve the lives of millions of women worldwide. AI is committed to the early diagnosis and treatment by automatically analyzing various imaging modes of breast cancer through large data sets of deep learning (61, 62). AI can reduce interval cancer in mammography screening (63). The accuracy of AI was higher than the elastic image and then than the conventional gray-scale image. With the assistance of the S-Detect AI system, the accuracy of BI-RADS classification was improved significantly (62). AI technology can automatically detect mitotic cells in histopathological images and accelerate the pathological diagnosis of breast cancer, including the multi-stage mitotic cell detection method of fast regional convolution neural network and deep CNN (64).

AI-based baseline molybdenum target and digital pathology to predict the response of breast cancer patients to neoadjuvant therapy can significantly improve the model for predicting NAC pathological response (47, 65).In addition, AI algorithm to analyze the prognosis of breast cancer patients is also an important development direction (66, 67).

The rising cost of cancer treatment has placed a financial burden on the health system. AAIC can reduce the cost of screening and the number of false positive and negative results, which avoids wasting the expenditure on treatment. The diagnostic accuracy in guiding genotype directed therapy was 97%, and the average time to start treatment was less than one day (68). CT combined with AI resulted in a negative incremental cost-effectiveness ratio (ICER) compared to CT only, showing lower costs and higher effectiveness (69). The lowest net cost savings using AI-informed management strategies are estimated at $72 per screened patient (70).

## Limitations

There are still some limitations to our study:

1. The literature searched was only English articles, which may have overlooked some of the literature.
2. The absence of extended search terms resulted in an incomplete literature search section.
3. AI has developed rapidly in recent years, so this study may have a short timeframe and needs to be updated regularly.

## Conclusion

This analysis provides a comprehensive overview of AI-related research conducted in the field of cancer, serving to help researchers, policy makers and practitioners to understand better the current development and practical implications of cancer-related AI research and to predict future trends. The United States has significantly contributed to the development of AAIC. AI plays a vital role in prediction, diagnosis and treatment. Predicting of cancer risk and prognosis may have significant scope for development in AAIC. Future AAIC research may be dedicated to optimizing AI calculation tools, elevating accuracy, and promoting AI.

## Data availability statement

The original contributions presented in the study are included in the article/supplementary material. Further inquiries can be directed to the corresponding authors.

## Author contributions

SX, YW, and P-fL contributed to the conception and formulation. P-mF and X-cC collected and analyzed the data. P-fL, KM, and Q-XM wrote the first draft. Writing-review and editing were prepared by G-xL. S-yD, and GXL contributed to data presentation supervision. All authors contributed to the article and approved the submitted version.

## Funding

Hainan Provincial key research and development Program (ZDYF2021SHFZ086).

## Acknowledgments

The author (GXL) wishes to acknowledge the financial support of the “Xiamen Health High-Level Talent Training Program”.

## Conflict of interest

The authors declare that the research was conducted in the absence of any commercial or financial relationships that could be construed as a potential conflict of interest.

## Publisher’s note

All claims expressed in this article are solely those of the authors and do not necessarily represent those of their affiliated organizations, or those of the publisher, the editors and the reviewers. Any product that may be evaluated in this article, or claim that may be made by its manufacturer, is not guaranteed or endorsed by the publisher.
